# On a Substitute for McMunn’s Elixir of Opium

**Published:** 1851-09

**Authors:** Eugene Dupuy

**Affiliations:** Pharmaceutist, New York


*On a Substitute for McMunn's Elixir of Opium.* By EUGENE DUPUY, Pharmaceutist, New York.—Within a few years the use of this preparation of opium has been much extended in the United States, through the medium of the press, as well as from the commendation of a numerous class of our practitioners, who found it to possess a sedative property which the ordinary Tincture of Opium does not possess in a similar way. Yet many amongst them reluctantly made use of it, from the fact that its mode of preparation was kept from the public, and that the usual abuse of such preparation, fostered by *directions for use without the need of medical aid,* by mothers, nurses, etc., was a great objection to its employment by that class of practitioners who want to *know, not only* what is the effect of the medicines they administer, but also, what are their component parts, and how they are prepared. Having such men among the physicians honoring my establishment with their custom, I have endeavored to prepare for their use, substitutes for some of the nostrums possessed of some efficacy. As a result of my endeavors, I will state that my substitute for McMunn's Elixir has been tested for about six years, and has been found to possess the sedative property peculiar to it, without any of the unpleasant effects attributed to Laudanum.

The late Dr. Smyth Rodgers, formerly Professor in the New York College of Pharmacy, during his painful illness, had frequent recourse to it, and even preferred it to McMunn's preparation, according to his attending physician's statements, although he had, at first, great reluctance to try anything else. An advantage in my substitute is, that its manipulation is exceedingly simple, and that a country physician having at hand the necessary ingredients, can prepare it as well as the most expert pharmaceutist. I prepare it as follows:

Opium - - - - ʒx.

Water, - - - - q. s.

Alcohol, 95 per ct. ℥iv.

The opium is to be made into a thin pulp with water; the mixture allowed to stand in a cool place 48 hours, then transferred into an elongated glass funnel containing filtering paper; a superstratum of water equivalent to the bulk of the whole mass is added. When 12 ounces of liquid have been filtered, the alcohol is added to the filtered solution.*

About two-thirds of the substance of the opium is contained in the solution; the residue consisting chiefly in resin, caoutchouc and narcotina, together with the ligneous matter. Consequently, my substitute is nothing more or less than an aqueous solution of opium, nearly free from narcotina, preserved by alcohol.

Various names could be devised for it, but as it is intended to represent an article already used under a popular name, perhaps the appellation of "Elixir of Opium" might be retained for it, if no other be suggested better adapted.—*American Journ. Pharm.*

[The unpleasant effects of ordinary tincture of opium when administered to certain patients have long since originated attempts to modify that preparation; witness the *denarcotized* laudanum, Battley's sedative solution, and the preparation suggested by the late Mr. Duhamel, (Amer. Journ. Pharm. vol. xviii. p. 16,) which last is almost identical with the "Elixir" of Mr. Dupuy. The latter, however, has the advantage in more completely exhausting the opium and in being less alcoholic when finished. In common with many others, we have given an occasional thought to the probable mode of preparing the so called "McMunn's Elixir of Opium." It contains meconate of morphia, and hence is prepared with *neutral* solvents, so as not to disturb the natural state of combination in which the morphia exists. In glancing over the long list of the constituents of opium with the view of singling out those to which the unpleasant effects of laudanum may be attributed, perhaps none are more obnoxious to suspicion than the odorous principle, resin, acid extractive, thebaina, and perhaps codeia, and narcotina to some extent, although O'Shaughnessy and others have shown that it is extremely doubtful whether the latter really possesses any disturbing quality of the kind. By the following process, a solution of opium can be made, deprived almost wholly of the principles it is desirable to avoid, and presenting the morphia in the form of its natural salt:

Take of Opium in powder, ten drachms (troy,)
" Ether,
" Alcohol, each; four fluid ounces,
" Water, a sufficient quantity.

Macerate the opium in half a pint of water for two days and ex-

* The proportion of opium is the same as that in Tinct. Opii of the U. S. P.

press; subject the dregs to two successive macerations, using six fluid ounces of water each time, with expression, mix and strain the liquors, evaporate to two fluid ounces, and agitate the liquid with the ether several times during half an hour. Then separate the ether by means of a funnel, evaporate the solution of opium to dryness, dissolve the extract in half a pint of cold water, pour the solution on a filter, and after it has passed wash the filter with sufficient water to make the filtrate measure 12 fluid ounces, to which add the alcohol and mix.

The same result was arrived at by first digesting the powdered opium in ether at several macerations, until it was exhausted, then drying and exhausting it with water. The aqueous solution was evaporated to dryness and then re-dissolved, filtered, etc., as in the above.

The ethereal liquid was evaporated at each instance :—that obtained directly from the opium yielded a brown crystalline extract, weighing 22 grains; whilst that resulting from washing the solution of opium, afforded acicular crystals and groups of larger crystals in stellated form, with a little brown extract-like matter around the edges, amounting to two grains, and having but little odor, and which exists in the elixir of Mr. Dupuy. These crystals are not reddened in the slightest degree by nitric acid, which dissolves them with a yellow color. In this treatment, the ether removes all that the water has dissolved of the thebaina, the meconin, a part at least of the codeia, the odorous principle, meconate of narcotine, and fatty matter. The ethereal extract obtained directly from the opium, contains nearly the whole of the odorous matter and fatty matter, besides the narcotine, free and combined. The evaporation to dryness, and re-solution in water, removes the ethereal odor, and separates a portion of acid resin and extractive. Landerer, in another part of this number, speaks of the nauseating and other unpleasant effects of the exhalations from poppy plantations during the collection of the opium. May not the odorous principle of opium have something to do with this effect, and may not the removal or loss of this in the so-called *denarcotized laudanum*, and in *old opium pills*, be at least partially the reason of their diminished tendency to produce nausea and headache? Mr. Redwood considers the "sedative liquor of Battley," to be an aqueous solution of opium evaporated to dryness to get rid of the acid resin, re-dissolved in water, and a small portion of spirit added to give it permanence.]—*Ed. Journ. Pharm.*

